# Sensitivity of Platinum‐Based Chemotherapy and Efficacy of Arsenic Trioxide‐Based Non‐Platinum Chemotherapy Following the Progression of PARPi Maintenance Therapy: A Real‐World Study

**DOI:** 10.1002/cam4.71208

**Published:** 2025-09-22

**Authors:** Rui Gou, Qi Xie, Haishan Ren, Hui Zhang, Tingting Wang, Ziyu Li, Zhenning Li, Ruichen Wang, Yingchao Yang, Xiaoyan Shen, Yi Li, Yue Wang, Lihui Wei, Xiaoping Li

**Affiliations:** ^1^ Department of Obstetrics and Gynecology Peking University People's Hospital Beijing China; ^2^ Department of Obstetrics and Gynecology The People's Hospital of Qitaihe Qitaihe China; ^3^ Department of Obstetrics and Gynecology The Fifth Affiliated Hospital of Zhengzhou University Zhengzhou China; ^4^ Department of Obstetrics and Gynecology The People's Hospital of Qihe County Dezhou China; ^5^ Department of Obstetrics and Gynecology Shijiazhuang People's Hospital Shijiazhuang China

**Keywords:** arsenic trioxide, chemotherapy resistance, ovarian cancer, PARPi

## Abstract

**Background:**

Cross‐resistance is observed between platinum and Poly (ADP‐ribose) polymerase inhibitors (PARPi). We aim to propose the definition of PARPi resistance and demonstrate the best therapeutic strategy for patients with PARPi resistance.

**Methods:**

A retrospective analysis was performed on patients diagnosed with epithelial ovarian cancer from October 2015 to November 2022. Patients were treated with PARPi for more than 6 months and received chemotherapy after progression.

**Results:**

Totally, 41 patients were enrolled, with 21 receiving PARPi for 6 to 12 months and 20 for more than 12 months. The median duration of PARPi was 12 months, and the median time to second progression (TTSP) was 3.45 months (range, 1.0–20.2 months). The Kaplan–Meier and Cox analysis revealed a significantly shorter TTSP for patients who received PARPi for more than 12 months compared to those for 6 to 12 months. After PARPi resistance, 34 (82.9%) received platinum‐based chemotherapy, with an overall response rate (ORR) of 26.5% (9/34). Seven patients (17.1%) received arsenic trioxide (ATO)‐based chemotherapy, with an ORR of 57.1% (4/7). During subsequent chemotherapy, 12/34 patients switched to ATO‐based chemotherapy due to progression, of which five cases were evaluated as effective (41.7%).

**Conclusion:**

PARPi resistance has a negative impact on the subsequent chemotherapy. The progression of the disease beyond 6 to 12 months should be considered as acquired resistance. Non‐platinum chemotherapy, such as ATO‐based combined sequential chemotherapy, may emerge as the preferred option for patients with PARPi resistance.

**Abbreviations**
ATO: arsenic trioxide
CR: complete response
DCR: disease control rate
ORR: objective response rate
PARPi: Poly (ADP‐ribose) polymerase inhibitors
PEG‐rhG‐CSF: pegylated recombinant human granulocyte colony‐stimulating factor
PFI: platinum‐free interval
PR: partial response
SD: stable disease
TTSP: the time to second progression

## 1 Introduction

Ovarian cancer is the most lethal gynecological cancer [1]. The initial treatment for ovarian cancer is cytoreductive surgery combined with chemotherapy. Although platinum‐based chemotherapy is the standard treatment for ovarian cancer, 85% of epithelial ovarian cancer patients still experience recurrence and drug resistance [2, 3].

In recent years, molecular targeted therapy has evolved rapidly. Multiple targeted drugs such as Poly (ADP‐ribose) polymerase inhibitors (PARPi) and angiogenesis inhibitors have achieved gratifying data in ovarian cancer [4]. These drugs prolonged the duration from the last platinum chemotherapy to disease recurrence, that is, the platinum‐free interval (PFI) [2]. However, malignant tumors are highly dynamic and evolutionarily heterogeneous entities. Under selective pressure, tumor cells with strong adaptability were successfully screened [5]. Both primary and acquired resistance led to the poor response of patients to PARPi. Given the potential cross‐resistance between platinum and PARPi, it is urgent to clarify whether acquired resistance to PARPi leads to reduced sensitivity and efficacy of subsequent platinum‐based chemotherapy [3, 6]. Our team has proposed a sequential chemotherapy regimen based on arsenic trioxide (ATO) + taxanes + 5‐fluorouracil + adriamycin ± bevacizumab. The effectiveness of ATO‐based sequential combined chemotherapy in platinum‐resistant recurrent epithelial ovarian cancer has been confirmed by a single‐center, open phase II clinical study [7]. However, the best therapeutic strategy for recurrence during PARPi maintenance therapy remains unknown.

Therefore, we utilized real‐world data from China to elucidate the risk factors for the failure of PARPi maintenance therapy. Additionally, we proposed a definition of acquired resistance to PARPi and explored the impact of PARPi resistance on subsequent therapy sensitivity, efficacy, and prognosis. Finally, we propose a potential benefit of a non‐platinum chemotherapy regimen to improve the current situation.

## 2 Methods

### 2.1 Study Design and Population

We retrospectively reviewed clinicopathological data in our hospital from patients newly diagnosed between October 2015 and November 2022. The inclusion criteria were as follows: (1) age ≥ 18 years; (2) histologically confirmed diagnosis of epithelial ovarian cancer, fallopian tube cancer, and primary peritoneal adenocarcinoma; (3) received maintenance therapy with PARPi in the Peking University People's Hospital for more than 6 months and received subsequent chemotherapy after disease recurrence; (4) received laboratory tests and imaging examinations in our hospital regularly. We excluded patients who did not receive subsequent therapy, missed important clinical data, refused follow‐up, or lacked follow‐up data. The regimen of subsequent therapy was selected based on the clinical evidence, past therapy history, adverse events, and personal requests. Furthermore, the decision to transition to ATO‐based therapy is contingent upon the progression of the disease. This study was approved by the Ethics Committee of Peking University People's Hospital (approval number: 2023PHB348‐001), and informed consent was obtained from the patients or their representatives for the use of their clinical data and tumor tissue specimens. The reporting of this study conforms to the STROBE statement [8].

### 2.2 Data Collection

The clinicopathological data from medical records included age, *BRCA1/2* mutation status, *TP53* mutation status, histology, FIGO stage, neoadjuvant chemotherapy, surgery, postoperative residual diseases, chemotherapy lines prior to PARPi, CA125 level prior to PARPi, type and duration of PARPi, subsequent therapy, progression data, and survival information. Missing information was supplemented through phone follow‐up. The last follow‐up occurred in July 2024.

### 2.3 Quantitative Variables

The disease response was evaluated in accordance with the Response Evaluation Criteria in Solid Tumors (RECIST 1.1) or GCIG CA125 response criteria [9]. PFS1 is defined as the duration from the initial diagnosis of cancer to recurrence during maintenance therapy. PFS2 is defined as the duration from the initiation of the ATO‐based chemotherapy following maintenance therapy to the time of next disease progression or death. The time to second progression (TTSP) is defined as the duration from the initiation of subsequent therapy (including platinum‐based chemotherapy and ATO‐based chemotherapy) following maintenance therapy to the time of next disease progression or death. For those who were alive without next disease progression at the study's cutoff date, PFS and TTSP were censored at their last follow‐up. Objective response rate (ORR) is defined as the proportion of patients who achieve complete response (CR) or partial response (PR). Disease control rate (DCR) is defined as the proportion of patients who achieve objective response (CR + PR) or stable disease (SD).

### 2.4 Statistical Analysis

All analyses were conducted using the SPSS version 22.0 software. Categorical variables were expressed as frequency and percentage, while continuous variables were represented by mean ± standard deviation (SD). Univariate and multivariate Cox regression analyses were employed to identify the factors influencing prognosis. Survival time was estimated using the Kaplan–Meier method, and differences between groups were evaluated using the log‐rank test. The count data were analyzed using the chi‐square test. *p* < 0.05 was considered statistically significant.

## 3 Results

### 3.1 Baseline Demographics and Clinical Features of the Enrolled Patients

A total of 41 patients were enrolled in this study. The baseline characteristics of the population are shown in Table 1. Patients' mean age at diagnosis was 55.7 years (range, 34–80). Among them, 11 (26.8%, 11/41) received neoadjuvant chemotherapy, and 15 (36.6%, 15/41) received hyperthermic intraperitoneal chemotherapy. Out of the 41 cases, 26 (63.4%, 26/41) achieved R0 after surgery, while 9 (22.0%, 9/41) had residual lesions. Among the patients undergoing genetic testing, 8 (27.6%, 8/29) harbored *BRCA1/2* mutations, while 19 (65.5%, 19/29) harbored *TP53* mutations.

**TABLE 1:** Clinicopathological characteristics of the study population (*N* = 41).

Clinical characteristics	*n*	%
***N***	41	
**Mean age ± SD (years)**	55.7 ± 10.3	
**Age at diagnosis (years)**		
< 56	20	48.8%
≥ 56	21	51.2%
**Primary site**		
Ovary	38	92.7%
Fallopian tube	2	4.9%
Primary peritoneal	1	2.4%
**Histology**		
Serous	40	97.6%
Clear cell	1	2.4%
**FIGO Stage**		
I	2	4.9%
II	3	7.3%
III	29	70.7%
IV	7	17.1%
**Neoadjuvant chemotherapy**		
Yes	11	26.8%
No	30	73.2%
**Postoperative residual disease status**		
R0	26	63.4%
R1	7	17.1%
R2	2	4.9%
NA	6	14.6%
**Lines of chemotherapy before PARPi**		
1	26	63.4%
≥ 2	15	36.6%
**PARPi received**		
Olaparib	16	39.0%
Niraparib	25	61.0%
**Gene mutation**		
*BRCA1/2* mt	8	19.5%
*TP53* mt	19	46.3%
NA	12	29.3%

Abbreviations: FIGO, International Federation of Gynecology and Obstetrics; Mt., mutation; NA, not available; WT, wild‐type.

**FIGURE 1:**
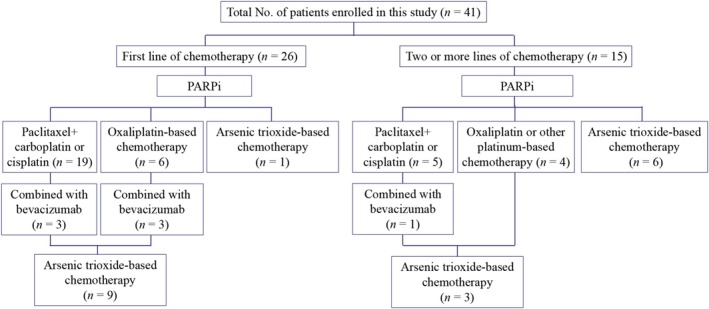
Details of therapies.

Details of therapies were shown in Figure 1. Before receiving PARPi, 26 (63.4%, 26/41) received first‐line chemotherapy, while 15 (36.6%, 15/41) received two or more lines of chemotherapy. All patients achieved CR or PR. Sixteen (39.0%, 16/41) and 25 (61.0%, 25/41) patients received olaparib and niraparib, respectively. The median duration of PARPi was 12 months (range, 6–29 months).

At follow‐up, all patients experienced a recurrence and received subsequent chemotherapy. Of these, 24 (58.5%, 24/41) received paclitaxel combined with carboplatin or cisplatin, 10 (24.4%, 10/41) received oxaliplatin‐based chemotherapy, and 7 (17.1%, 7/41) received ATO + taxanes + 5‐fluorouracil ± bevacizumab ± PD‐1 inhibitor sequential chemotherapy. During the subsequent chemotherapy, 12 out of 34 patients receiving platinum‐based chemotherapy switched to ATO‐based chemotherapy due to disease progression. Among them, 10 to 15 mg per day of ATO was given via intravenous drip for a total of 7 to 8 days.

### 3.2 Influencing Factors for PFS

The duration of progression from diagnosis to maintenance therapy was analyzed for survival (Table 2). Univariate analysis showed that FIGO stage (HR, 3.909; 95% CI, 1.351–11.312; *p* = 0.012), residual disease (HR, 2.031; 95% CI, 1.032–3.997; *p* = 0.040), *TP53* mutation status (HR, 4.852; 95% CI, 1.575–14.946; *p* = 0.006), lines of chemotherapy before PARPi (HR, 0.421; 95% CI, 0.206–0.859; *p* = 0.018) were associated with PFS1. No significant impact was found in age, value of pretreatment CA‐125, neoadjuvant chemotherapy, hyperthermic intraperitoneal chemotherapy, *BRCA1/2* mutation status, the number of first‐line chemotherapy, CA‐125 before PARPi, and type of PARPi (all *p* > 0.05). Multivariate analysis showed that *TP53* mutation was an independent risk factor for poor PFS1 (HR, 6.280; 95% CI, 1.625–24.267; *p* = 0.008).

**TABLE 2:** Univariate and multivariate Cox regression analyses of risk factors for PFS1.

	Univariate analysis		Multivariate analysis	
	HR (95% CI)	*p*	HR (95% CI)	*p*
Age (years) (≥ 56 vs. < 56)	1.729 (0.899–3.326)	0.101		
Pretreatment CA‐125 (U/mL) (≥ 1455^a^ vs. < 1455)	0.830 (0.377–1.826)	0.644		
Neoadjuvant chemotherapy (yes vs. no)	1.691 (0.823–3.474)	0.153		
HIPEC (yes vs. no)	1.050 (0.526–2.095)	0.890		
FIGO stage (III–IV vs. I–II)	3.909 (1.351–11.312)	0.012*	4.139 (0.865–19.813)	0.075
FIGO stage (IV vs. III)	0.796 (0.321–1.976)	0.623		
Residual disease (≥ R1 vs. R0)	2.031 (1.032–3.997)	0.040*	1.878 (0.896–3.937)	0.095
*BRCA* mutation (yes vs. no)	1.133 (0.501–2.560)	0.764		
*TP53* mutation (yes vs. no)	4.852 (1.575–14.946)	0.006*	6.280 (1.625–24.267)	0.008*
The number of first‐line chemotherapy (> 6 vs. ≤ 6)	1.070 (0.536–2.135)	0.848		
Lines of chemotherapy before PARPi (≥ 2 vs. 1)	0.421 (0.206–0.859)	0.018*	1.052 (0.368–3.007)	0.925
CA‐125 before PARPi (U/mL) (≥ 35 vs. < 35)	0.946 (0.352–2.546)	0.913		
Type of PARPi (Niraparib vs. Olaparib)	1.438 (0.750–2.757)	0.274		

Abbreviations: FIGO, International Federation of Gynecology and Obstetrics; HIPEC, hyperthermic intraperitoneal chemotherapy.

^a^ Median value of pretreatment CA125.

* *p* < 0.05.

### 3.3 Impact of PARPi Duration on the Efficacy of Subsequent Chemotherapy

To date, all patients experienced a recurrence during PARPi maintenance therapy and received subsequent chemotherapy. Of the 41 patients, 21 (51.2%, 21/41) received PARPi for 6 to 12 months, while 20 for more than 12 months (48.8%, 20/41). The median TTSP was 3.45 months (range, 1.0–20.2 months). To clarify the impact of the duration of PARPi maintenance therapy on the efficacy of subsequent chemotherapy, all patients were divided into two groups based on the duration of 12 months.

**FIGURE 2:**
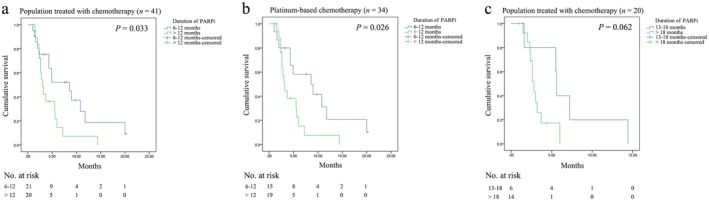
Kaplan–Meier curves of the time to second progression. (a) Analysis of all enrolled patients. (b) Analysis of patients receiving platinum‐based chemotherapy. (c) Analysis of patients receiving PARPi for more than 12 months.

The Kaplan–Meier analysis revealed that patients with a duration of PARPi maintenance for more than 12 months exhibited a significantly poorer TTSP compared to those with maintenance for 6 to 12 months (*p* = 0.033; Figure 2a). After excluding seven patients using ATO‐based chemotherapy, this conclusion also applies to patients receiving platinum‐based chemotherapy (*p* = 0.026; Figure 2b). Further dividing patients who received PARPi for more than 12 months into two groups based on a cutoff of 18 months, no statistically significant difference was found in the impact of 13 to 18 months and more than 18 months on TTSP (*p* = 0.062; Figure 2c). Hence, a 12‐month duration of maintenance therapy can be considered a threshold for indicating subsequent chemotherapy resistance.

The Cox analysis showed that the duration of PARPi exceeding 12 months was a risk factor affecting TTSP (HR, 2.230; 95% CI, 1.045–4.759; *p* = 0.038). However, TTSP was not significantly correlated with FIGO stage, lines of chemotherapy before PARPi, type of PARPi, serum CA‐125 levels before subsequent chemotherapy, subsequent chemotherapy regimens, combination with immunotherapy or antiangiogenic therapy, and genetic mutation status (all *p* > 0.05; Table 3). Therefore, the duration of PARPi is an important factor influencing TTSP.

**TABLE 3:** Cox regression analysis of risk factors for TTSP.

	Univariate analysis	
	HR (95% CI)	*p*
Duration of PARPi (> 12 months vs. 6–12 months)	2.230 (1.045–4.759)	0.038*
FIGO stage (III–IV vs. I–II)	1.156 (0.437–3.061)	0.770
Lines of chemotherapy before PARPi (≥ 2 vs. 1)	0.833 (0.365–1.899)	0.663
Type of PARPi (Niraparib vs. Olaparib)	0.690 (0.331–1.440)	0.323
CA‐125 before subsequent chemotherapy (U/mL) (≥ 70 vs. < 70)	1.205 (0.389–3.729)	0.746
Subsequent chemotherapy (carboplatin or cisplatin vs. oxaliplatin)	0.543 (0.234–1.259)	0.155
Subsequent chemotherapy combined with immunotherapy or antiangiogenic therapy (yes vs. no)	0.647 (0.234–1.723)	0.384
*BRCA* mutation (yes vs. no)	0.705 (0.274–1.817)	0.470
*TP53* mutation (yes vs. no)	1.498 (0.576–3.895)	0.407

Abbreviation: FIGO, International Federation of Gynecology and Obstetrics.

* *p* < 0.05

### 3.4 Impact of PARPi Resistance on the Efficacy of Platinum‐Based and Non‐Platinum‐Based Chemotherapy

To assess the optimal chemotherapy following PARPi resistance, we conducted further analysis to evaluate the efficacy of subsequent chemotherapy. The overall evaluation of 34 patients receiving platinum‐based chemotherapy yielded the following results: it has been found that 3, 6, 9, and 16 patients (8.8%, 17.6%, 26.5%, and 47.1%) were in CR, PR, SD, and PD, respectively. The ORR and DCR were 26.5% (9/34) and 52.9% (18/34) respectively. Seven patients who received ATO‐based chemotherapy following PARPi resistance demonstrated an ORR of 57.1% (4/7).

During the subsequent chemotherapy, 12/34 patients switched to ATO‐based chemotherapy due to disease progression. The median number of cycles for ATO‐based chemotherapy was four times (range, 1–10 times). Among the cases, five were deemed effective based on imaging or tumor biomarkers (41.7%, 5/12), and three were classified as SD (25.0%, 3/12). Based on the aforementioned data, a total of 19 patients received ATO‐based chemotherapy. The total ORR was 47.4% (9/19), which was higher than the ORR of patients receiving platinum‐based chemotherapy (26.5%, 9/34).

**FIGURE 3:**
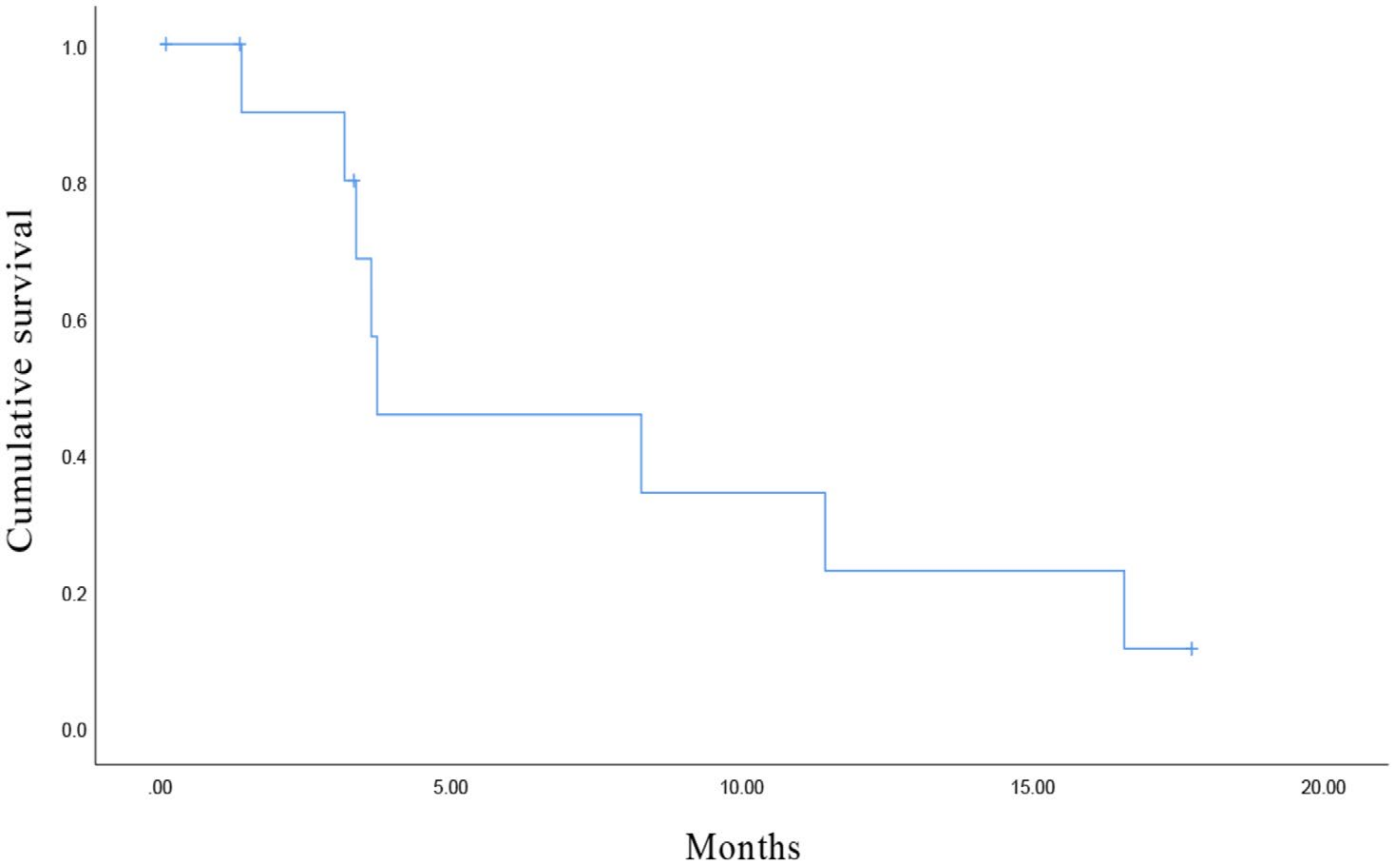
Kaplan–Meier curves of the PFS2 from initiation of ATO‐based chemotherapy in the 12 patients who changed regimens due to progression.

By the end of the follow‐up, the median PFS2 from initiation of ATO‐based chemotherapy in the 12 patients who changed regimens due to progression was 3.5 months (range, 0.10–17.73 months, Figure 3). This cohort of patients underwent one or two lines of platinum‐based chemotherapy prior to the ATO‐based chemotherapy. In sum, the median duration from initiation of subsequent chemotherapy following PARPi to disease progression or death during ATO‐based chemotherapy in these patients was 12.05 months (range, 6.13–18.97 months). Till now, among all the patients who have received ATO‐based chemotherapy, two of them successfully completed ATO‐based chemotherapy and subsequently received niraparib again, while another patient received anlotinib following ATO‐based chemotherapy.

Most adverse events were grade 1 to 2. The most frequently reported adverse event was myelosuppression (100% of patients), among which 31.6% were grade 3 or above. Due to previous febrile neutropenia, preventive injection of pegylated recombinant human granulocyte colony‐stimulating factor (PEG‐rhG‐CSF) in some patients may affect this result. In addition, nausea (73.7%), constipation (47.4%), drug‐induced liver damage (42.1%), and fatigue (26.3%) were also relatively common. None of the patients developed perforations, fistulas, hypersensitivity, or treatment‐related deaths.

## 4 Discussion

PARPi resistance leads to a poor prognosis for patients. Based on factors such as tumor heterogeneity and genetic mutations, PARPi resistance can be categorized as either primary resistance or acquired resistance [10]. Currently, research on PARPi resistance is predominantly at the basic research stage. Distinguishing acquired resistance in the early stage from primary resistance in clinical settings remains challenging. This study utilizes real‐world data from China to explore the definition of clinical resistance to PARPi, elucidate the impact of PARPi on subsequent chemotherapy, and investigate the efficacy of ATO‐based combined sequential non‐platinum chemotherapy for patients with PARPi resistance. These findings provide guidance and assistance to clinical practice.

### 4.1 Definition, Duration and Classification of PARPi Resistance, and Its Impact on Sensitivity to Subsequent Chemotherapy

The PFI plays an indispensable role in guiding clinical chemotherapy [11]. According to PFI length (< 1 month, 1–6 months, 6–12 months, and > 12 months), recurrent ovarian cancer was categorized into platinum‐refractory, platinum‐resistant, potentially platinum‐sensitive, and fully platinum‐sensitive [11, 12]. However, the precise definition of platinum‐resistant recurrence has become a clinical challenge due to interfering factors such as targeted drugs for maintenance therapy. Similarly, the definition of PARPi resistance in clinical practice remains to be clarified.

To exclude the influence of primary resistance, this study enrolled patients who received PARPi for more than 6 months. The real‐world data demonstrated that TTSP in patients with a duration of PARPi for more than 12 months was significantly shorter than those for 6 to 12 months. When the time duration of maintenance therapy with PARPi before recurrence exceeds 12 months, it indicates a high probability of resistance to subsequent chemotherapy. Therefore, we propose that the progression of the disease beyond 6 to 12 months should be considered as acquired resistance.

What is the rationale behind PARPi's impact on the sensitivity of platinum‐based chemotherapy? The mechanisms of PARPi resistance mainly include the restoration of HR repair, dysregulation of multiple signaling pathways, stabilization of the replication fork, and abnormal transmembrane transport [13]. Of concern is the overlap of these mechanisms with platinum resistance. For example, reversion mutations or epigenetic alterations leading to the re‐expression of a BRCA1 or BRCA2 wild‐type protein or resulting in hypomorphic variants are common resistance mechanisms to PARPi, which is also the main mechanism for platinum resistance in *BRCA1/2*‐mutated cancer [14]. Considering cross‐resistance mechanisms, resistance to PARPi may potentially impact the sensitivity to subsequent chemotherapy.

The first clinical evidence of a potential adverse effect of PARPi on subsequent platinum‐based chemotherapy came from the post hoc analyses of SOLO2. Compared with placebo, patients receiving olaparib as maintenance therapy showed diminished efficacy to subsequent platinum‐based chemotherapy, with TTSP of 14.3 and 7 months, respectively [15]. After analyzing 66 *BRCA* mutated patients who were treated with subsequent chemotherapy after olaparib progression, MITO found that the ORR for patients with a PFI of more than 12 months and those with an interval of 6 to 12 months was only 22.2% and 11.1%, respectively [16]. Patients with platinum‐sensitive *BRCA1/2* mutated tumors who progressed during olaparib maintenance following second‐line chemotherapy exhibited a reduced likelihood of responding to third‐line chemotherapy compared to controls who did not receive olaparib [17]. However, conclusions of different studies may vary due to varying inclusion criteria and interfering factors. Another study showed that for patients who had received two or more lines of chemotherapy before PARPi maintenance therapy, no negative effects of PARPi on subsequent therapy were found, regardless of *BRCA* status. In addition, for 65 platinum‐sensitive patients, no negative impact on subsequent chemotherapy was observed in relation to the duration of PARPi maintenance on TTSP in the multivariate regression analysis [18]. A study conducted in Japan revealed that the use of bevacizumab in subsequent therapies was identified as an independent prognostic factor for PFS, while the duration of PARPi maintenance did not show any correlation with PFS [19]. However, this study had a limited sample size, with only 18 patients receiving platinum‐based chemotherapy after PARPi. Furthermore, 88.9% of the enrolled patients received PARPi for a duration ranging from 6 to 12 months. The discrepancy in PARPi duration may impact the results. Therefore, it is difficult to draw unambiguous conclusions from such a small number of patients.

### 4.2 Which is Superior: Platinum‐Based Chemotherapy or Non‐Platinum‐Based Chemotherapy Following PARPi Resistance?

Currently, platinum‐based drugs remain the cornerstone of chemotherapy for recurrent ovarian cancer. A multicenter study focuses on the results of subsequent platinum‐based regimens in the population remaining platinum‐sensitive after progression to PARPi. *BRCA*‐mutant patients presented significantly higher rates of progression (45.7% vs. 17.9%, respectively) and worse survival outcomes (mPFS, 3.5 m vs. 7.5 m; mOS 16.4 vs. 24.2 m) under subsequent platinum than non‐*BRCA*‐mutant patients [20]. It suggested that previous PARPi may result in *BRCA* reversal mutations, potentially compromising the efficacy of subsequent platinum‐based regimens in *BRCA* mutant patients. Recently, a real‐world study proposed that for patients progressing on PARPi with a PFI > 6 months, platinum‐based chemotherapy rechallenge is the best option [21]. However, the therapy period in this study spans from 2003 to 2021. Due to the approval policy regarding PARPi, only 14.1% of patients were treated with PARPi in the first‐line setting. The maximum duration of PARPi exposure reached 54.3 months, which may differ from current clinical practices. In addition, the primary regimen for patients undergoing non‐platinum‐based chemotherapy typically involves paclitaxel monotherapy. The suboptimal therapeutic efficacy associated with this single‐agent strategy may contribute to discrepancies in research outcomes. The results of our study indicated that the ORR for patients receiving platinum‐based chemotherapy after PARPi resistance was only 26.5%, suggesting that PARPi resistance has a negative impact on the efficacy of subsequent chemotherapy. What is the optimal chemotherapy regimen for patients with PARPi resistance?

Surprisingly, we confirmed that the ORR of patients receiving ATO‐based combined sequential non‐platinum chemotherapy was 57.1%. Arsenic‐containing compounds have been used in traditional Chinese medicine for more than 2000 years and are widely used as first‐line therapies for acute promyelocytic leukemia. As a wide‐spectrum cytotoxic and highly immunogenic drug, ATO has been proven to be effective in the treatment of solid tumors [22, 23]. Research has confirmed that ATO exerts its anti‐cancer effects by regulating biological processes, such as cell apoptosis, ferroptosis, oxidative stress, angiogenesis, cell cycle arrest, and cancer stem cell stemness [24, 25]. Existing research indicates that ATO in combination with olaparib activated AMPK α‐SCD1 signaling, thereby inducing ferroptosis in platinum‐resistant ovarian cancer cells and suppressing tumor growth in mice xenograft models [26]. Based on the synergistic effect of inducing DNA damage and apoptosis, ATO sensitized homologous recombination proficient ovarian cancer to PARPi [27]. In addition, ATO induces cytotoxicity and apoptosis in cisplatin‐sensitive and ‐resistant ovarian cancer cell lines [28, 29]. Therefore, the prospects of ATO in drug‐resistant ovarian cancer are promising. Our team conducted a single‐center, open phase II clinical study and confirmed that the ORR of platinum‐resistant recurrent epithelial ovarian cancer patients receiving ATO‐based chemotherapy was 42%, with a PFS of 9.5 months and an OS of 16.2 months. Immune checkpoint inhibitors exert antitumor effects by activating immune response. The ORR of monotherapy with PD‐1/PD‐L1 inhibitors in recurrent ovarian cancer ranges from 4% to 15% [30]. Chemotherapy can activate effector cells, inhibit immunosuppressive cells, or enhance immunogenicity, thereby enhancing the infiltration of immune cells and augmenting the efficacy of immunotherapy [30]. A multicenter phase II clinical study in our country has confirmed the efficacy of sintilimab combined with bevacizumab in relapsed or persistent ovarian clear cell carcinoma [31]. Another study confirmed that ATO enhanced the efficacy of PD‐1 inhibitors in hepatocellular carcinoma by inducing immunogenic cell death [32]. Therefore, in this study, some patients who received ATO‐based chemotherapy were combined with PD‐1 inhibitors. Similar to our study, the main side events included myelosuppression, minor gastrointestinal, and liver toxicities [7]. Therefore, ATO‐based chemotherapy is effective for recurrent drug‐resistant patients.

This study confirms that 41.7% of patients showed effectiveness when switching to ATO‐based chemotherapy after progression on subsequent platinum‐based chemotherapy. Furthermore, the PFS of this cohort has been further prolonged. Due to the different mechanisms of ATO, taxanes, fluorouracil, bevacizumab, and PD‐1 inhibitors, this sequential chemotherapy regimen overcomes the problem of cross‐resistance and may become a recommended non‐platinum‐based chemotherapy for PARPi‐resistant patients, especially for those using PARPi for more than 12 months. On the other hand, it can be used as an alternative option after the progression of platinum‐based chemotherapy following PARPi therapy.

This study has several limitations. Although we have previously confirmed the efficacy of ATO‐based chemotherapy in ovarian cancer, this approach has not been widely adopted. Therefore, the number of cases in this study is limited. Secondly, due to the long time span, genetic testing data for some patients is unavailable, which may lead to biased results in the analysis of this subset. Additionally, due to economic reasons, most patients did not undergo HRD testing. Lastly, some patients are still undergoing subsequent chemotherapy and therefore mature OS data is not available. The conclusion needs to be further verified with long‐term follow‐up and large sample sizes.

## 5 Conclusions

In conclusion, this study utilizes real‐world data to demonstrate that PARPi resistance has a negative impact on the sensitivity of subsequent chemotherapy. We preliminarily established a time point of 12 months as the most appropriate cutoff for PARPi resistance. When the duration of maintenance therapy with PARPi before disease progression exceeds 12 months, it indicates a high probability of resistance to subsequent platinum‐based chemotherapy. Due to the presence of cross‐resistance, platinum‐based chemotherapy may no longer be the preferred option for patients who are resistant to PARPi. The utilization of non‐platinum chemotherapy, such as ATO‐based combined sequential chemotherapy, has demonstrated certain effects. Further validation is needed through multicenter studies.

## Author Contributions

**Rui Gou:** writing – original draft, writing – review and editing, resources, data curation, conceptualization. **Qi Xie:** writing – review and editing, resources, data curation. **Haishan Ren:** data curation, resources, writing – review and editing. **Hui Zhang:** data curation, resources, writing – review and editing. **Tingting Wang:** data curation, resources, writing – review and editing. **Ziyu Li:** writing – review and editing, formal analysis, data curation. **Zhenning Li:** data curation, formal analysis, writing – review and editing. **Ruichen Wang:** data curation, formal analysis, writing – review and editing. **Yingchao Yang:** writing – review and editing, resources. **Xiaoyan Shen:** writing – review and editing, project administration, methodology. **Yi Li:** methodology, writing – review and editing, project administration. **Yue Wang:** writing – review and editing, project administration, methodology. **Lihui Wei:** project administration, methodology, writing – review and editing. **Xiaoping Li:** writing – review and editing, resources, data curation, project administration, conceptualization.

## Ethics Statement

This study protocol was reviewed and approved by the Ethics Committee of Peking University People's Hospital, approval number [2023PHB348‐001].

## Consent

Written informed consent was obtained from participants.

## Conflicts of Interest

The authors declare no conflicts of interest.

## Data Availability

The data that support the findings of this study are available from the corresponding author upon reasonable request.
